# Defect‐Free Sb‐Doping in Bi_2_O_2_Se Achieves Two‐Order‐of‐Magnitude Reduction in Saturation Intensity While Preserving High Carrier Mobility

**DOI:** 10.1002/advs.202518454

**Published:** 2025-11-19

**Authors:** Qingling Tang, Zhongben Pan, Hongwei Chu, Han Pan, Dechun Li

**Affiliations:** ^1^ School of Information Science and Engineering, and Key Laboratory of Laser and Infrared System of Ministry of Education Shandong University Qingdao 266237 China

**Keywords:** carrier mobility, first principles calculation, low saturation intensity, Sb‐doped Bi_2_O_2_Se, ultrafast pulse generation

## Abstract

Doping generally introduces performance trade‐offs in materials, yet overcoming this fundamental limitation remains crucial for advancing materials research. Bi_2_O_2_Se exhibits exceptional electronic properties as a promising semiconductor, yet its nonlinear optical response under low excitation intensities hinders its practical applications. Therefore, precise Sb^3^⁺ doping in Bi_2_O_2_Se (Bi_1.9_Sb_0.1_O_2_Se) is achieved for the first time via solid‐state reaction and systematically studies its impact on the electronic structure and optical properties through first‐principles calculations and experimental. The results reveal that Sb^3^⁺ substitution slightly reduces the bandgap without introducing defect states, and transient absorption spectroscopy further confirms prolonged carrier relaxation. At 1.5 µm, the modulation depth from 8.8% to 10.1% while dramatically reducing the saturation intensity from 47.2 to 0.53 kW cm^−^^2^. This improvement is attributed to the stable linear absorption characteristics after doping, the synergistic effect between prolonged relaxation time and free‐carrier‐induced optical loss. In a mode‐locking system, Bi_1.9_Sb_0.1_O_2_Se achieves a broader 3‐dB and shorter pulse duration at substantially reduced pump intensities. This work achieves defect‐free energy level optimization in Sb‐doped Bi_2_O_2_Se, where the material's high carrier mobility is not only preserved but further enhanced, while the saturation intensity is declined by about two orders of magnitude, enabling a low‐power, high‐performance nonlinear photonic devices.

## Graphical abstract

This study demonstrates defect‐free states Sb^3^⁺ doping in Bi_2_O_2_Se, enhancing carrier mobility while reducing saturation intensity by about two orders of magnitude. First‐principles calculations and experiments reveal optimized electronic structure and improved nonlinear response. The doped material enables low‐threshold mode‐locked lasers with broader bandwidth and shorter pulses, advancing energy‐efficient nonlinear photonic devices.

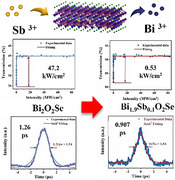

## 1 Introduction

In materials science, performance modulation often encounters a fundamental challenge—the inherent trade‐off between different material properties. Taking graphene as an example, its gapless characteristic endows exceptionally high carrier mobility, yet the introduction of a bandgap for achieving high on/off ratios leads to a significant mobility degradation, a contradiction that limits its electronic applications.^[^ ^1^ ^]^ Similarly, nitrogen doping in MXenes significantly enhances electrochemical performance by introducing redox‐active sites, increasing electron density, and expanding interlayer spacing to improve ion transport kinetics; however, this doping process simultaneously induces structural modifications that may ultimately compromise the material's structural stability and cycling durability.^[^ ^2^ ^]^ Such trade‐offs are also observed in thermoelectric materials, as exemplified by p‐type doped a chalcogenide perovskite CaZrSe_3_ where the Seebeck coefficient exhibits an inverse relationship with carrier mobility.^[^ ^3^ ^]^ Therefore, exploring strategies to synergistically optimize target properties while preserving intrinsic material advantages represents a highly valuable research direction in materials science.

In recent years, the emerging layered semiconductor material Bi_2_O_2_Se has garnered widespread attention due to its unique tetragonal crystal structure (space group *I*4/mmm) and exceptional charge transport properties.^[^ ^4^, ^5^, ^6^ ^]^ The material features an alternating stacking of positively charged [Bi_2_O_2_]^2^⁺ layers and negatively charged [Se]^−2^ layers, where each oxygen atom coordinates with four bismuth atoms to form Bi_4_O tetrahedral structural units.^[^ ^7^ ^]^ This structure spatially separates the electron conduction channels from donor sites, where the [Bi_2_O_2_]^2^⁺ layers play the role of conducting channel in Bi_2_O_2_Se, while the donor sites (such as Se vacancies) are located in the Se layer, thereby significantly suppressing electron carrier scattering.^[^ ^8^ ^]^ The Shubnikov‐de Haas (SdH) oscillation measurements reveal that Bi_2_O_2_Se exhibits Fermi velocities comparable to graphene,^[^ ^9^, ^10^ ^]^ along with pronounced small‐angle forward scattering and suppressed electron backward scattering, which collectively contribute to its ultrahigh electron mobility.^[^ ^11^, ^12^, ^13^ ^]^ In addition, Bi_2_O_2_Se exhibits enormous potential for realizing optical modulators due to its ultrabroadband nonlinear optical response.^[^ ^14^ ^]^

Saturation intensity serves as a critical performance parameter for optoelectronic devices, with its optimization holding substantial implications across multiple applications. In STED nanoscopy, reduced saturation intensity (fluorescence carbon dots: 0.226 MW·cm^−2^, CsPbBr_3_ quantum dots: 0.126 MW cm^−2^) enables lower depletion beam power requirements while maintaining imaging resolution.^[^ ^15^, ^16^ ^]^ For ultrafast lasers, this characteristic facilitates lower operational thresholds in mode‐locked systems, promoting the development of compact, low‐power femtosecond sources.^[^ ^17^, ^18^ ^]^ In addition, experimental and theoretical studies have confirmed that semiconductor saturable absorber mirrors (SESAMs) can achieve a significant reduction in saturation intensity from the 1 MW/cm^2^ to 3.45 kW cm^−^^2^ range through low‐doping strategies.^[^ ^19^ ^]^ However, SESAMs still face limitations, including high cost, narrow operational bandwidth, and complex fabrication processes.^[^ ^20^ ^]^ Based on these findings, we propose precisely controlling doping concentrations to optimize the light‐matter interaction strength in materials. Our primary objective is to retain high carrier mobility while reducing saturation intensity through rational doping selection. This approach aims to achieve faster saturation responses at lower optical intensities while circumventing the technical bottlenecks of conventional SESAM devices, thereby providing a more universal design strategy for developing novel saturable absorber materials.

Building upon this foundation, we successfully synthesized Sb‐doped Bi_2_O_2_Se with a chemical composition of Bi_1.9_Sb_0.1_O_2_Se through solid‐state reactions (verified by ICP‐MS). First‐principles calculations revealed, for the first time, the electronic‐level mechanism involving charge redistribution and band structure modulation induced by Sb substitutional doping in Bi_2_O_2_Se, demonstrating that Sb doping not only reduces the bandgap but also avoids introducing defect states, thereby preserving the material's high carrier mobility. Transient absorption spectroscopy (TAS) results confirm that Sb doping prolongs the carrier relaxation time, indicating suppressed nonradiative recombination and enhanced carrier accumulation. I‐scan showed that the doped material achieved modulation depths of 8.8% and 10.1% at 1.5 µm wavelength, with corresponding saturation intensities of 47.2 and 0.53 kW cm^−^^2^, representing approximately two orders of magnitude reduction compared to the undoped counterpart. To validate practical applicability, Bi_2_O_2_Se and Bi_1.9_Sb_0.1_O_2_Se are connected to Erbium‐doped fiber lasers (EDFL), achieving stable mode‐locking operation and conventional soliton spectrum output. These results not only confirm the remarkable efficacy of Sb‐doped Bi_2_O_2_Se in reducing saturation intensity but also directly demonstrate its practical utility in ultrashort pulse generation through mode‐locked laser experiments, thereby offering a novel material solution for developing high‐performance optoelectronic devices.

## 2 Results and Discussion

### 2.1 Morphological Characterization and Elemental Analysis

**Figure 1:**
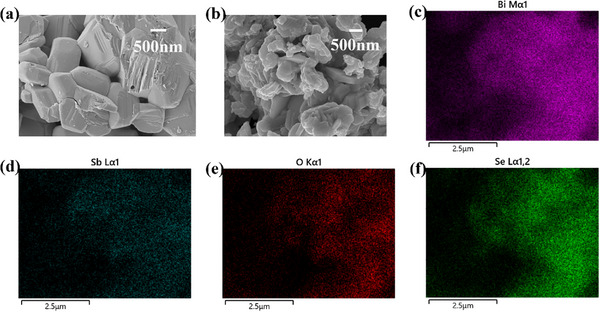
a) SEM of Bi_2_O_2_Se b) SEM of Bi_1.9_Sb_0.1_O_2_Se c–f) EDS element mapping image of Bi, Sb, O, and Se in Bi_1.9_Sb_0.1_O_2_Se.

The morphology and microstructure of Bi_2_O_2_Se before and after doping were characterized by scanning electron microscopy (SEM). As shown in **Figure** **1**a, the undoped sample exhibits highly crystalline, regular block‐like or columnar crystals with large grain sizes (average >500 nm), featuring distinct crystal facets and edges, where particles are densely packed with tight intergranular bonding. In contrast, the doped sample (Figure 1b) demonstrates significant morphological changes, the crystal structure becomes looser and more disordered, with markedly reduced grain sizes (mostly <200 nm), displaying thinner and more flaky. This morphological evolution provides direct evidence for structural defects introduced by doping.

To accurately determine the Bi:Sb stoichiometric ratio in Bi_2_O_2_Se and characterize its elemental distribution, we conducted a systematic analysis using multi‐scale characterization techniques. First, ICP‐MS was employed for bulk composition analysis (Table S1, Supporting Information), revealing a Bi:Sb molar ratio of 1.9:0.1 (Bi_1.9_Sb_0.1_O_2_Se). To further verify the uniformity of elemental distribution, energy‐dispersive spectroscopy (EDS) elemental mapping (Figure 1c–f) was performed, demonstrating that Bi and Sb exhibit atomic‐scale homogeneous distribution without observable segregation.

### 2.2 Structural Characterization

**Figure 2:**
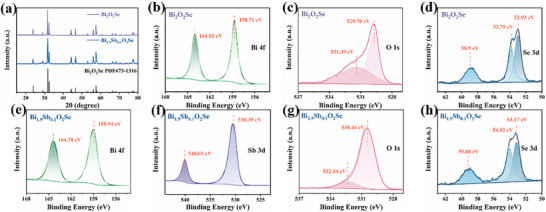
a) XRD of Bi_2_O_2_Se and Bi_1.9_Sb_0.1_O_2_Se, b)‐d) XPS of Bi_2_O_2_Se, e)‐h) XPS of Bi_1.9_Sb_0.1_O_2_Se.

As shown in **Figure** **2**a, the undoped sample exhibits multiple diffraction peaks at 3.86°, 29.10°, 31.68°, 32.42°, 44.18°, 46.58°, 53.06°, 56.08°, 57.50°, 66.36°, 68.08°, and 77.36°, which perfectly match the standard reference PDF#73‐1316 (Bi_2_O_2_Se), confirming the high crystallinity and phase purity of the sample. After Sb doping, all diffraction peaks systematically shift to higher angles (29.14°, 31.72°, 32.48°, 44.30°, 46.64°, 53.12°, 56.12°, 57.60°, 66.44°, 68.18°, 77.48° etc.), directly indicating lattice contraction. This shrinkage primarily results from the equivalent substitution of Bi^3^⁺ (1.03 Å) by smaller Sb^3^⁺ (0.76 Å). The slight but distinct peak shifts provide direct structural evidence for effective Sb doping and maintaining the structural integrity of the material. Notably, a weak diffraction peak appears at 2θ = 30.76°, identified as the BiSbO_4_ secondary phase (PDF#89‐2008), which aligns with previous reports of possible byproducts in Sb‐doped Bi_2_O_2_Se systems.^[^ ^21^ ^]^ To further investigate the impact of this impurity, we specifically synthesized BiSbO_4_ samples (XRD shown in Figure S1, Supporting Information) and, for the first time, conducted nonlinear optical property measurements on this compound in subsequent studies.

To further evidence the effectiveness of Sb doping and the chemical state of the elements of Bi_1.9_Sb_0.1_O_2_Se, we systematically investigated the surface chemical states of Bi_2_O_2_Se and Bi_1.9_Sb_0.1_O_2_Se using X‐ray photoelectron spectroscopy (XPS) in Figure 2b–h. For the undoped sample, the Bi 4*f* orbital exhibited a characteristic doublet structure with peaks at 158.71 and 164.02 eV, while the Se 3*d* orbital showed peaks at 52.93, 53.79 and 58.9 eV. The O 1*s* orbital displayed characteristic peaks at 529.7 eV (lattice oxygen) and 531.39 eV (surface‐adsorbed oxygen), with all binding energy values perfectly matching the standard chemical states of Bi_2_O_2_Se.^[^ ^22^, ^23^ ^]^ In the Bi_1.9_Sb_0.1_O_2_Se, the XPS spectra revealed significant changes: all core levels (Bi 4f, Se 3d, and O 1s) exhibited a systematic shift of ≈0.23–0.95 eV toward higher binding energies, indicating electron density redistribution induced by Sb incorporation. Additionally, characteristic peaks of Sb 3d appeared at 530.39 eV (Sb 3d5/2) and 54.03 eV (Sb 3d3/2), with binding energies consistent with the Sb^3^⁺ oxidation state, further confirming the successful incorporation of Sb into the Bi_2_O_2_Se lattice at Bi sites.

### 2.3 Theoretical Calculation

**Figure 3:**
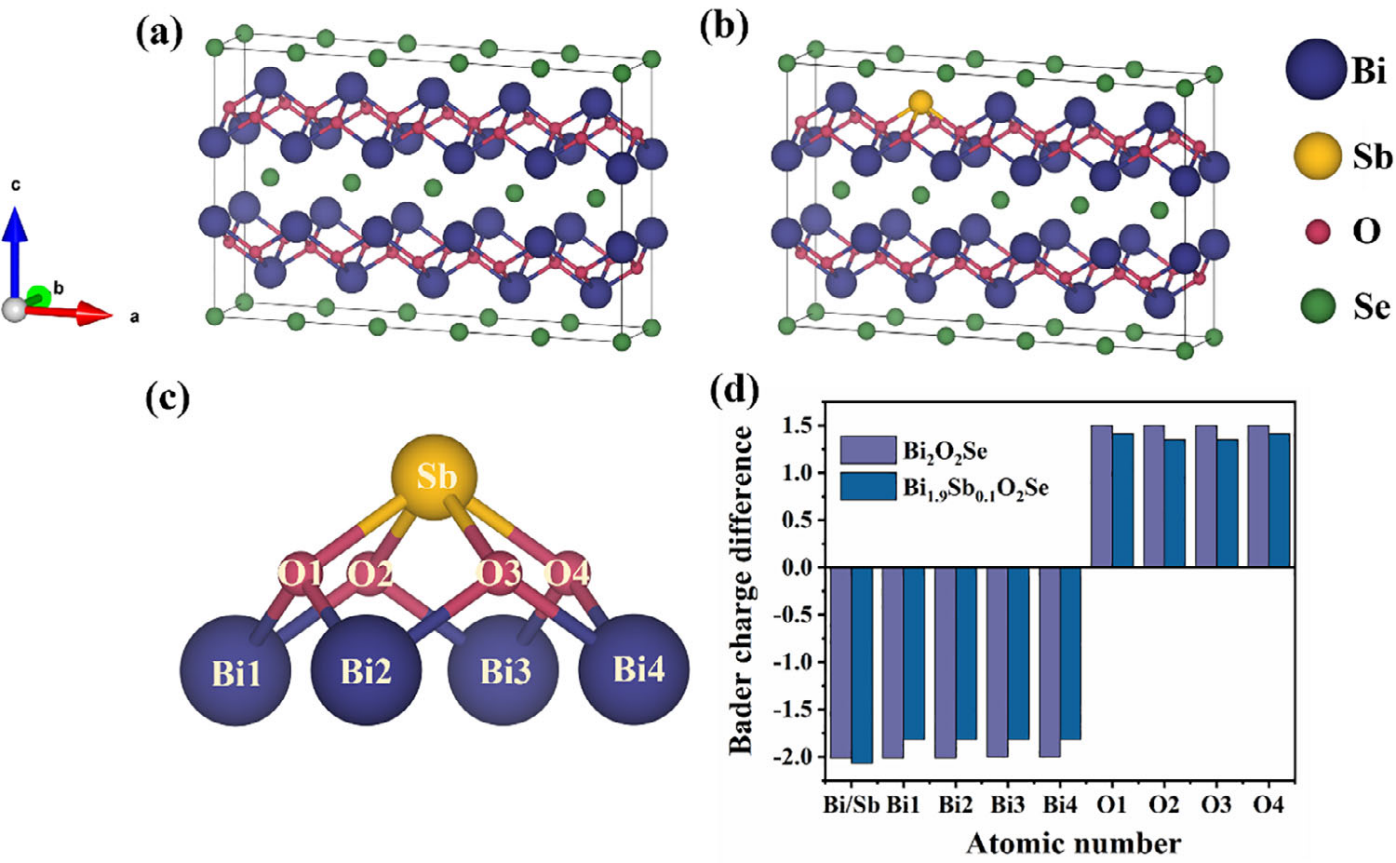
a) Crystal structure of Bi_2_O_2_Se, b) Crystal structure of Bi_1.9_Sb_0.1_O_2_Se, c) Doping site crystal structure, d) bader charge difference (The horizontal axis corresponds to the crystal structure illustrated in (c)).

**Figure 4:**
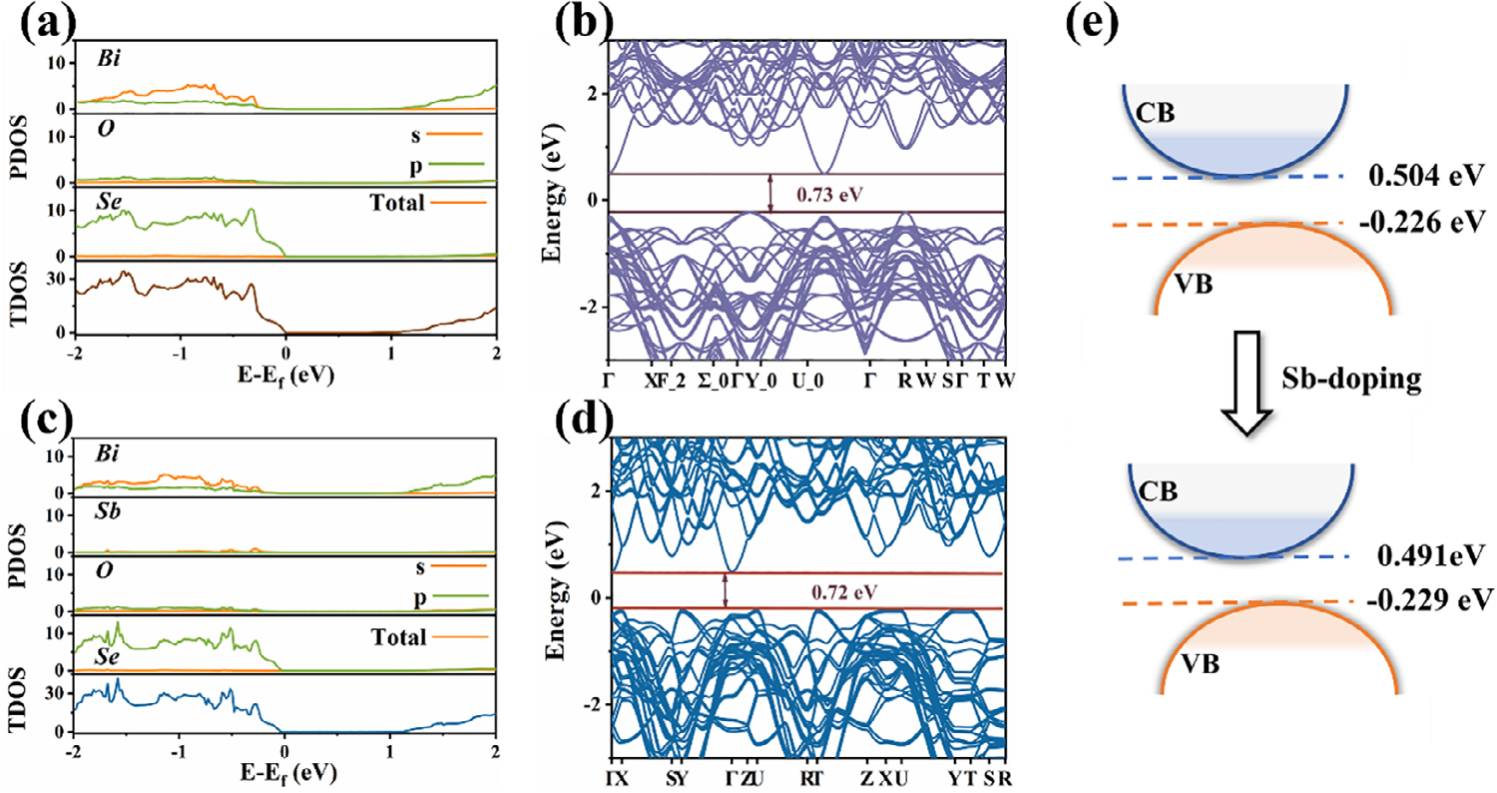
a) Dos of Bi_2_O_2_Se, b) band structure of Bi_2_O_2_Se, c) Dos of Bi_1.9_Sb_0.1_O_2_Se, d) band of Bi_1.9_Sb_0.1_O_2_Se, e) Schematic diagram of bandgap renormalization shrinkage.

To elucidate the microscopic mechanism of Sb‐doped Bi_2_O_2_Se, we conducted systematic structural analysis based on first‐principles calculations. To accurately simulate the doping concentration of experimentally synthesized samples, a 5 × 1 × 1 supercell model was employed (**Figure** **3**a). Given the equivalence of all Bi sites in Bi_2_O_2_Se, one Bi site was selectively substituted with Sb to construct a doped model with the chemical formula Bi_1.9_Sb_0.1_O_2_Se (Figure 3b). The structural optimization reveals distinct bond length variations around the doping sites: the average Sb‐O bond length contracted by 0.175 Å, while the average Bi‐O bond length elongated by 0.06 Å (Table S2, Supporting Information). Bader charge analysis reveals that compared to the original Bi atoms, the doped Sb atoms lose more charge, which can be attributed to the higher tendency of Sb (5s^2^5p^2^) to donate electrons compared to Bi (6s^2^6p^3^) (Figure 3d). Their coordinated O atoms gain less charge, and the adjacent Bi atoms exhibit reduced charge depletion. This localized charge redistribution directly influences the electronic structure. The density of states (DOS) analysis (**Figure** **4**a,c) reveals that the valence band maximum (VBM) remains predominantly governed by Se‐4*p* orbitals, while the conduction band minimum (CBM) is primarily dominated by Bi‐6*p* orbitals. The evolution of the band structure demonstrates that doping induces a significant downward shift of the CBM and a slight downward shift of the VBM, resulting in a bandgap reduction of 0.01 eV without introducing defect states (Figure 4b,d).

This behavior can be attributed to the interplay of electronic and structural effects: 1) The Sb dopant introduces additional electrons into the conduction band, increasing the occupancy of Bi's density of states and lowering the CBM energy—consistent with Bader charge analysis showing reduced charge loss from Bi. 2) The slight downward shift of the VBM is induced by the weak perturbation of Sb substitution for Bi on the Se‐dominated states. The similar radius and same valence state of Sb and Bi can minimize lattice distortion and prevent the introduction of defect states near VBM. Therefore, the narrowing of the bandgap is mainly driven by conduction band filling (CBM offset), rather than by valence band modification or defect state formation (Figure 4e), demonstrating a controllable strategy for band engineering in optoelectronic applications.

**Figure 5:**
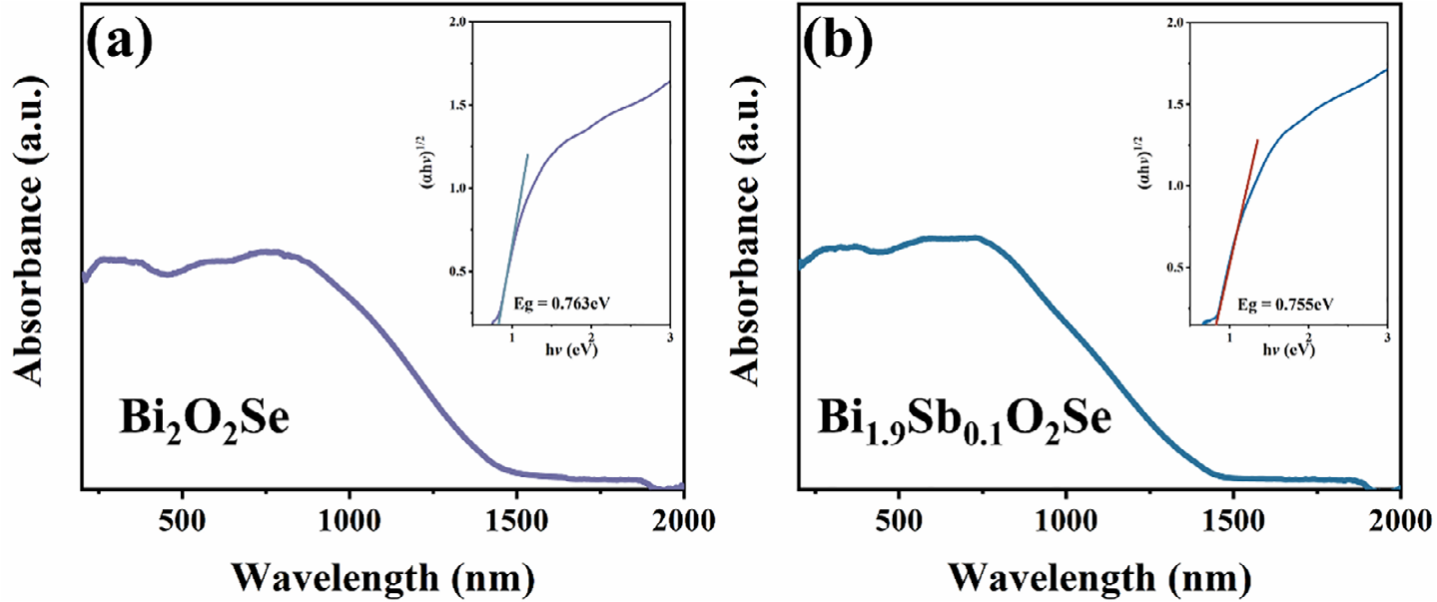
a) UV−VIS−IR absorption spectra of Bi_2_O_2_Se, inset: Dependence of (𝛼hv)^1/2^ on photon energy h*v*, and b) UV−VIS−IR absorption spectra of Bi_1.9_Sb_0.1_O_2_Se, inset: Dependence of (𝛼hv)^1/2^ on photon energy h*v*.

Furthermore, the bandgap of the material using ultraviolet‐visible‐near infrared (UV‐VIS‐NIR) absorption spectroscopy (in **Figure** **5**). Tauc plot analysis revealed that the optical bandgap of Bi_2_O_2_Se decreased by ≈0.008 eV after Sb doping. This trend aligns well with the band structure evolution predicted by first‐principles calculations, and the consistency between experiment and theory further validates the reliability of Sb doping‐induced band narrowing in modulating the electronic structure. This small bandgap reduction likely originates from the synergistic effects of localized lattice distortion (e.g., shortened Sb─O bonds) and charge redistribution on the band‐edge states, ultimately improving carrier mobility and reducing saturated intensity. This is consistent with existing experimental conclusions.^[^ ^21^ ^]^ Additionally, UV‐VIS‐NIR measurements and bandgap fitting of the BiSbO_4_ material (Figure S2, Supporting Information) indicate a significantly wider bandgap (2.6 eV) with an absorption edge far below that of Bi_2_O_2_Se. Thus, it can be inferred that trace amounts of BiSbO_4_ would not noticeably affect the absorption characteristics of the Sb‐doped samples.

### 2.4 Femtosecond‐Resolved Transient Absorption Spectroscopy of Bi_2_O_2_Se and Bi_1.9_Sb_0.1_O_2_Se

**Figure 6:**
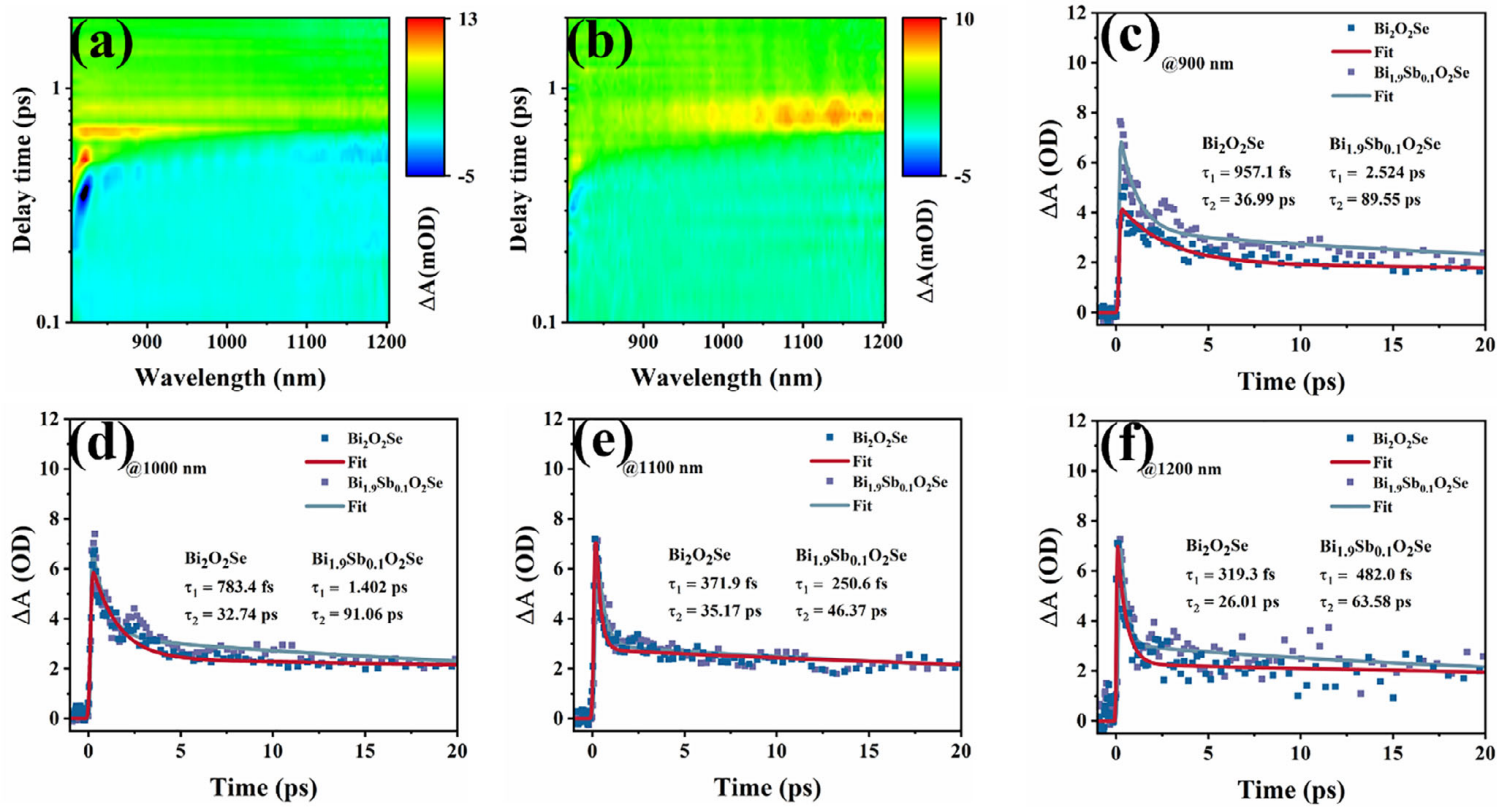
a) Pseudo‐color diagram of TAS for Bi_2_O_2_Se, b) Pseudo‐color diagram of TAS for Bi_1.9_Sb_0.1_O_2_Se. The transient absorption curves of Bi_2_O_2_Se and Bi_1.9_Sb_0.1_O_2_Se at the probe wavelength are c) 900 nm, d) 1000 nm, e)1100 nm, and f) 1200 nm.

To gain insight into the carrier dynamics and their modulation by Sb incorporation, femtosecond transient absorption spectroscopy (TAS) was employed to probe the ultrafast relaxation processes in Bi_2_O_2_Se and Bi_1.9_Sb_0.1_O_2_Se. The pseudo‐color diagrams of both Bi_2_O_2_Se and Bi_1.9_Sb_0.1_O_2_Se (**Figure** **6**a,b) exhibit similar spectral features, namely, a ground‐state bleaching (ΔA < 0) centered ≈800 nm and a pronounced photoinduced absorption (ΔA > 0) extending beyond 1000 nm. However, the extracted kinetic traces at characteristic probe wavelengths (900–1200 nm, Figure 6c–f) reveal slower decay dynamics in the Sb‐doped sample, indicating prolonged carrier relaxation and extended lifetime upon Sb incorporation. Global analysis using a biexponential decay model yields average lifetimes of τ₁ = 1.16 ps and τ_2_ = 72.64 ps for Bi₁.₉Sb₀.₁O_2_Se, which are significantly longer than those of pristine Bi_2_O_2_Se (τ₁ = 607.93 fs, τ_2_ = 32.73 ps). The biexponential decay function can be expressed as follows ^[^ ^24^ ^]^

(1) $$\Delta A=A_{1}\exp\left(-\frac{t}{\tau_{1}}\right)+A_{2}\exp\left(-\frac{t}{\tau_{2}}\right)$$

where A_1_ and A_2_ are the first and second amplitude terms, t is the delay time, and τ_1_, τ_2_, represent the fast and slow response times, respectively. Importantly, first‐principles calculations confirm that Sb incorporation does not introduce mid‐gap states but instead leads to a moderate bandgap narrowing in Bi_2_O_2_Se. This modification slightly increases the density of states near the band edges and reduces the density of deep‐level defects. Consequently, the carrier relaxation observed in TAS becomes slower, reflecting suppressed nonradiative recombination and extended carrier lifetime. Meanwhile, the reduced defect scattering facilitates higher carrier mobility in the equilibrium state.

### 2.5 Doping Mediated Nonlinear Optical Properties

**Figure 7:**
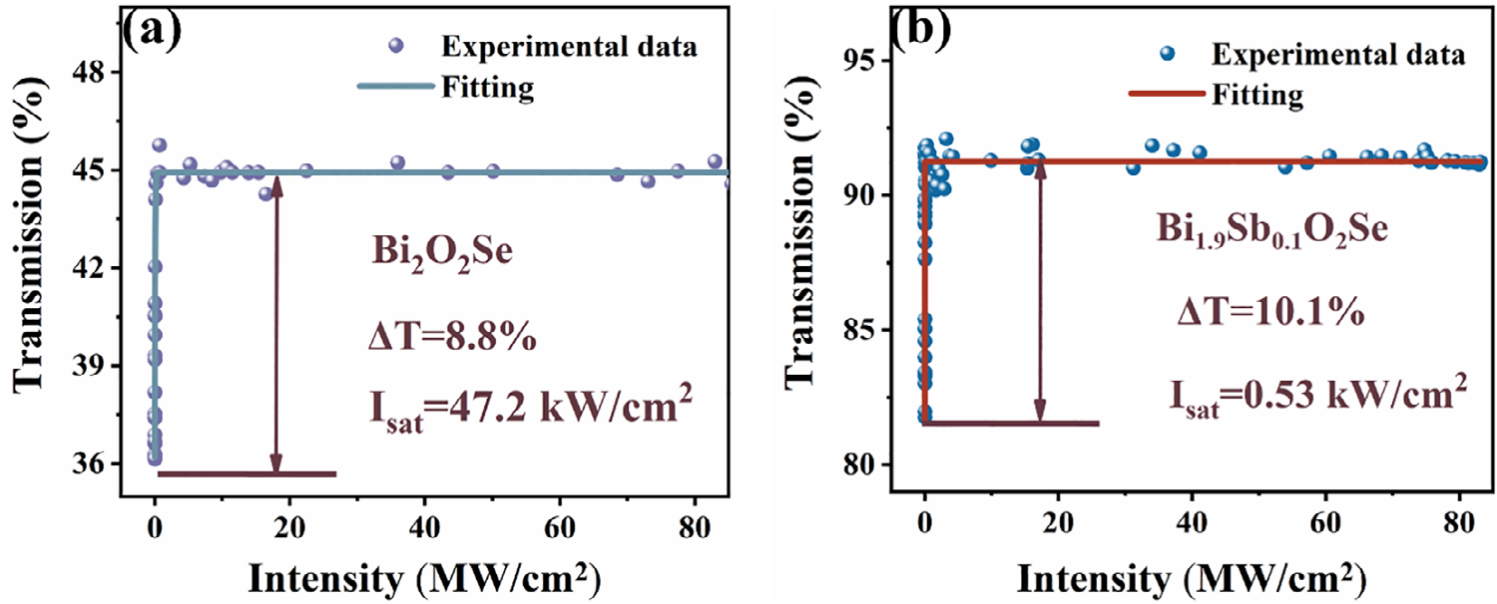
The nonlinear transmittance curves of a) Bi_2_O_2_Se and b) Bi_1.9_Sb_0.1_O_2_Se at 1.5 µm.

New saturable‐absorption materials exhibiting low saturation intensity are critical and have become an important research target for ultrafast optoelectronics. To investigate the influence of Sb^3^⁺ incorporation on the nonlinear optical properties of Bi_2_O_2_Se, a dual‐balanced detection system was specifically designed, as illustrated in Figure S3 (Supporting Information). **Figure** **7** presents the nonlinear transmittance versus incident light intensity for Bi_2_O_2_Se and Bi_1.9_Sb_0.1_O_2_Se in the 1.5 µm range, measured using this dual‐balanced detection system. At lower incident intensities, the samples exhibit relatively low transmittance, which gradually increases with intensity until reaching a plateau, demonstrating saturable absorption behavior. The relationship between transmittance and incident intensity was fitted using the following equation ^[^ ^25^, ^26^ ^]^

(2) $$T=1-\Delta Texp\left(-\frac{I}{I_{s}}\right)-T_{ns}$$

where *T* is the transmittance, Δ*T* is the modulation depth, *I_s_* represents the saturation intensity, and *T_ns_* is the non‐saturable loss. *I_s_* And Δ*T*are fundamental parameters for predicting whether a material is suitable for fiber lasers. The fitting results reveal that for Bi_2_O_2_Se at 1.5 µm, Δ*T* = 8.8% and *I_s_* = 47.2 kW cm^−^^2^ (Figure 7a). Under the same experimental conditions, the Bi_1.9_Sb_0.1_O_2_Se exhibits a higher modulation depth (Δ*T* = 10.1%) and a significantly lower saturation intensity (*I_s_* = 0.53 kW cm^−^^2^) at 1.5 µm (Figure 7b). Additionally, I‐scan measurements were performed on BiSbO_4_, yielding Δ*T* of 6.7% and *I_s_* of 1.74 kW cm^−^^2^ (Figure S4, Supporting Information). In comparison, both ∆T and *I_s_* of BiSbO_4_ are inferior to those of Bi_1.9_Sb_0.1_O_2_Se. This indicates that Sb doping (rather than forming BiSbO_4_) more effectively optimizes the saturable absorption properties of Bi_2_O_2_Se. Notably, Sb‐doped Bi_2_O_2_Se exhibits a higher modulation depth alongside a significantly reduced saturation intensity.

We attribute the observed significant reduction in saturation intensity to the modulation of Bi_2_O_2_Se by Sb doping. As described by the typical expression for saturation intensity is ^[^ ^27^ ^]^:

(3) $$I_{s}=\frac{hv}{\sigma \tau }$$

where *hv* is photon energy, σ is the absorption cross section, σ is the absorber recovery time. Based on first‐principles calculations and experimental characterizations, Sb doping does not significantly alter the intrinsic bandgap of Bi_2_O_2_Se (only reducing it by ≈0.01 eV), and no new peaks appear in the linear absorption spectrum at the wavelength of 1.5 µm, indicating negligible changes in photon energy and absorption cross section under this excitation wavelength. However, the absorber recovery time is a macroscopic response parameter whose variation is governed by multiple physical mechanisms, including the material's relaxation time. Our transient absorption spectroscopy study revealed that Sb doping prolongs the carrier relaxation time. This can be attributed to the effective introduction of additional charge carriers by Sb doping, which aligns well with the increased carrier concentration reported in the literature. ^[^ ^21^ ^]^ Due to the free‐carrier absorption coefficient depends linearly on the carrier concentration,^[^ ^28^ ^]^ the enhanced carrier concentration further amplifies free‐carrier absorption, leading to non‐radiative dissipation of incident optical energy through electron‐phonon coupling. Therefore, we attribute the observed phenomenon to the synergistic effect between prolonged relaxation time and free‐carrier‐induced optical loss, while the intrinsic linear absorption characteristics remain stable. This ultimately reduces the saturation intensity of the material from 47.2 to 0.53 kW cm^−^^2^ (small‐scale nonlinear transmittance curves in Figure S5, Supporting Information), a decrease of nearly two orders of magnitude.

### 2.6 The Improvement of Pulsed Laser Performance

**Figure 8:**
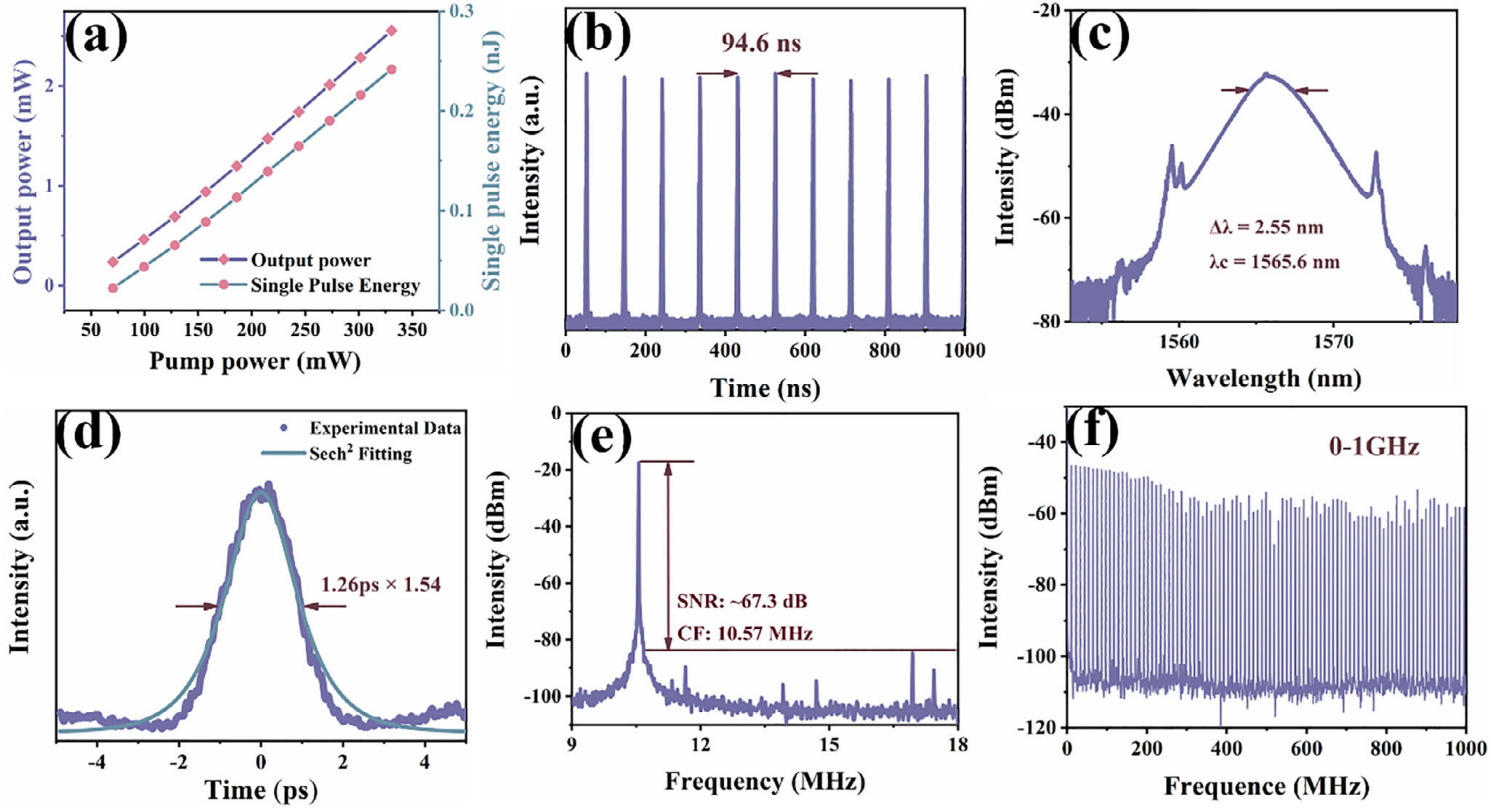
The mode‐locking pulse output characteristics of Bi_2_O_2_Se at 1.5 µm. a) Variation of output power and single pulse energy. b) Pulse sequence. c) Optical spectrum. d) Autocorrelation trajectory. e) RF spectrum. f) RF spectrum within a span of 0–1 GHz.

To further investigate the ultrafast response characteristics of Bi_2_O_2_Se before and after modification, the samples were integrated into a fiber ring cavity to explore their mode‐locking laser performance. Figure S6 (Supporting Information) illustrates the experimental setup of a mode‐locked fiber laser using Bi_2_O_2_Se as the SA. When a tapered fiber (diameter: 18.4 µm, insertion loss: 10%) without SA material deposition was introduced into the laser cavity, only continuous‐wave (CW) light was generated regardless of pump power or polarization controller (PC) adjustments. However, after depositing Bi_2_O_2_Se onto the tapered fiber and integrating it into the EDFL ring cavity, conventional soliton mode‐locking operation was achieved at a pump power of 301.58 mW by fine‐tuning the PC, as shown in **Figure** **8**. Figure 8a displays the linear relationship between output power, single‐pulse energy, and pump power. As the pump power increased from 70.37 to 330.49 mW, the output power rose from 0.24 to 2.56 mW, corresponding to a single‐pulse energy increase from 0.02 pJ to 0.24 nJ. Figure 8b shows a typical pulse train with a 1 µs time span and a pulse interval of 94.6 ns, matching a total cavity length of 19.4 m. Figure 8c presents the conventional soliton mode‐locking spectrum centered at 1565.6 nm, with a 3 dB spectral width of 2.55 nm. The presence of distinct Kelly sidebands confirms the laser's operation in the conventional soliton mode‐locking regime. The spectral asymmetry can be attributed to Raman self‐frequency shift, soliton‐dispersive wave interactions, and asymmetric gain spectra.^[^ ^29^ ^]^ As shown in Figure 8d, hyperbolic secant fitting of the autocorrelation trace yielded a pulse width of 1.26 ps. The calculated time‐bandwidth product (TBP) of 0.393 slightly exceeds the theoretical limit for transform‐limited pulses (0.315), indicating minor chirping. Figure 8e shows the radio frequency (RF) spectrum with a central frequency of 10.57 MHz and a signal‐to‐noise ratio (SNR) of ≈67.3 dB. Figure 8f further demonstrates the robust stability of the conventional soliton mode‐locking achieved with the Bi_2_O_2_Se SA, as evidenced by the RF spectrum over a 1 GHz span.

**Figure 9:**
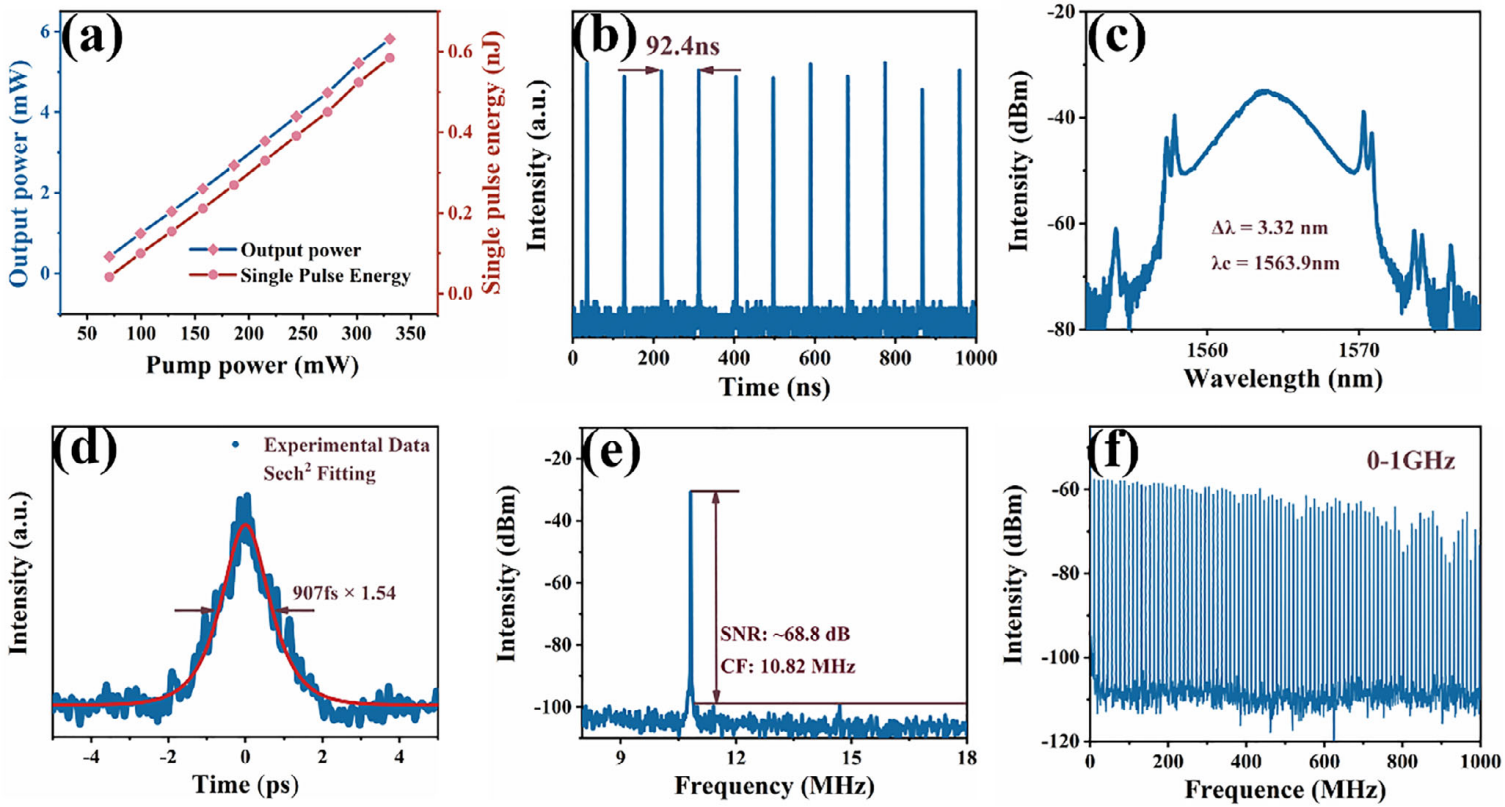
The mode‐locking pulse output characteristics of Bi_1.9_Sb_0.1_O_2_Se at 1.5 µm. a) Variation of output power and single pulse energy. b) Pulse sequence. c) Optical spectrum. d) Autocorrelation trajectory. e) RF spectrum. f) RF spectrum within a span of 0–1 GHz.

Similarly, the tapered fiber with Bi_1.9_Sb_0.1_O_2_Se SA (diameter: 17.2 µm, insertion loss: 9.5%) was integrated into an EDFL cavity. By gradually increasing the pump power and carefully adjusting the intracavity polarization state, conventional soliton mode‐locking was achieved at a pump power of 157.08 mW, this threshold power is significantly lower than that required for mode‐locking with Bi_2_O_2_Se, further confirming that the material's reduced saturation intensity enables mode‐locking operation at substantially lower pump powers. **Figure** **9**a reveals a linear increase in output power (0.42 to 5.82 mW) and single‐pulse energy (0.04 pJ to 0.58 nJ) as the pump power was raised from 70.37 to 330.49 mW. Figure 9b shows a pulse train with a 1 µs span and a 92.4 ns interval, corresponding to a cavity length of 19.0 m. The mode‐locking spectrum (Figure 9c) centered at 1563.9 nm exhibited a 3 dB bandwidth of 3.32 nm, with clear Kelly sidebands confirming soliton formation. Autocorrelation fitting gave a pulse width of 907 fs (Figure 9d), and the TBP of 0.369 indicated slight chirping. The RF spectrum (Figure 9e) had a central frequency of 10.82 MHz and an SNR of ≈68.8 dB, while Figure 9f verified the stability over a 1 GHz span. To further verify the optimization effect of Sb doping on mode‐locking performance, comparative tests were carried out using BiSbO_4_, which is also the first time this material has been applied in mode‐locking research (see Figure S7, Supporting Information). The results revealed that it exhibited inferior performance compared to Bi_1.9_Sb_0.1_O_2_Se, with a narrower 3 dB bandwidth (2.4 nm) and a broader pulse width (1.25 ps), further confirming the enhancing effect of Sb doping.

Comparative analysis shows that lower saturation intensity and higher modulation depth in Sb doping achieve wider 3 dB bandwidth and shorter pulse width, further verifying the excellent nonlinear optical performance of Bi_1.9_Sb_0.1_O_2_Se. Besides, the performances of different types of material SAs and SESAMs based mode‐locked operations are listed in **Table** **1**. Compared to conventional semiconductor SAs and SESAM devices, Bi_2_O_2_Se, BiSbO_4_, and Bi_1.9_Sb_0.1_O_2_Se all exhibit superior nonlinear optical responses and mode‐locking performance, among them, Bi_1.9_Sb_0.1_O_2_Se exhibits an extremely low saturation intensity, indicating that it can reach the saturated absorption state under relatively low optical intensity, which is advantageous to achieve mode‐locking.^[^ ^30^ ^]^ Their fiber lasers outperform most semiconductor‐based systems in key metrics such as bandwidth and pulse width, providing a promising material platform for high‐performance mode‐locked lasers.

**Table 1:** Performance Summary of Mode‐Locking Fiber Lasers Operating in and EDFL by Using Different Types of Materials as SAs.

Material	Modulation Depth (%)	Saturation Intensity (MW/cm^2^)	Center Wavelength (nm)	3 dB Δλ (nm)	Pulsed Width (ps)	Refs.
CuO	5.8	20.8	1565.8	2.2	1.26	[31]
Bismuthene	7.7	16	1530.4	2.8	1.75	[32]
SESAM	15	0.15	1561	1.16	3.86	[33]
Fe_3_O_4_	7	141	1572.39	1.39	2.93	[34]
In_2_Se_3_	3.8	246.6	1529.4	3.96	1.38	[35]
Cr_2_Sn_2_Te_6_	6.17	15.97	1530.1	3.55	1.26	[36]
Co_3_O_4_	13	8.3	1558.86	2.25	1.24	[37]
γ‐MnO_2_	‐	‐	1561	3.4	0.968	[38]
rGO‐ZIF‐67	6.61	28	1529.8	1.9	2.44	[39]
Bi_2_O_2_Se	8.8	0.047	1565.6	2.55	1.26	This work
BiSbO_4_	6.7	0.0017	1564.1	2.40	1.25	This work
Bi_1.9_Sb_0.1_O_2_Se	10.1	0.00053	1563.9	3.32	0.907	This work

## 3 Conclusion

In summary, this study successfully incorporated Sb into Bi_2_O_2_Se via a solid‐state reaction, with ICP characterization confirming the chemical composition as Bi_1.9_Sb_0.1_O_2_Se. Combining first‐principles calculations and experimental studies, we systematically elucidated the mechanism of Sb doping's influence on electronic structure and optoelectronic properties. VASP calculations with Bader charge analysis revealed that Sb doping reduces lattice dimensions, decreases charge depletion in Bi atoms, and induces local charge redistribution. Density of states and band structure analyses demonstrated that the doped system retains the Se‐4p‐dominated VBM and Bi‐6p‐dominated CBM, with the CBM shifting downward by 0.013 eV and VBM slightly decreasing by 0.003 eV, resulting in a 0.01 eV bandgap reduction without introducing defect states—consistent with the 0.008 eV optical bandgap reduction measured via Tauc plot. Transient absorption spectroscopy further reveals that Sb incorporation results in slower carrier relaxation dynamics, reflecting a reduction in nonradiative recombination pathways and more efficient carrier transport due to the decreased defect scattering. Consequently, This defect‐free bandgap engineering enhances carrier mobility. I‐scan measurements showed that Sb doping increases modulation depth from 8.8% to 10.1% while reducing saturation intensity by two orders of magnitude (from 47.2 to 0.53 kW cm^−^^2^). Analysis using the classical saturation intensity expression revealed nearly unchanged linear absorption characteristics, with prolonged relaxation time leading to increased recovery time of the saturable absorber and enhanced free‐carrier‐induced optical loss due to higher carrier concentration—these mechanisms collectively contribute to the reduced saturation intensity. When implemented in a mode‐locked laser, Bi_1.9_Sb_0.1_O_2_Se achieved stable mode‐locking at only 50% of the pump power required for Bi_2_O_2_Se, while demonstrating superior performance with broader 3‐dB bandwidth and shorter pulse duration. The simultaneous optimization of carrier mobility and saturation light intensity provides a novel material design strategy for high‐performance near‐infrared optoelectronic devices.

## 4 Experimental Section

### Synthesis of Bi_2_O_2_Se Powder

Bi_2_O_3_ and Bi_2_Se_3_ were thoroughly mixed in a 2:1 molar ratio and loaded into a corundum crucible. The crucible was then placed in a chemical vapor deposition (CVD) system, which was repeatedly flushed with high‐purity argon to ensure an inert atmosphere. Argon was introduced as a carrier gas at a flow rate of 100 sccm. The system was heated at a ramp rate of 10 °C/min to a target temperature of 600 °C, held for 2 h to ensure complete reaction, and then cooled naturally to room temperature. The final black product obtained after thermal treatment was characterized and used for all subsequent studies.

### Synthesis of Bi_1.9_Sb_0.1_O_2_Se Powder

Bi_2_O_3_, Bi_2_Se_3,_ and Sb_2_Se_3_ were mixed in a 11:4:1 molar ratio and processed similarly. The CVD reaction was conducted at 630 °C for 2 h under the same argon flow conditions (100 sccm), followed by natural cooling. The final black product obtained after thermal treatment was characterized and used for all subsequent studies.

### Synthesis of BiSbO_4_ Powder

Bi_2_O_3_ and Sb_2_O_3_ were mixed in a 1:1 molar ratio, heated at 600 °C for 12 h, and then slowly cooled to room temperature at a rate of 1 °C/min. The resulting product was reground, heated again at 850 °C for 24 h, and cooled to room temperature at the same rate (1 °C/min). All heating processes were conducted in air, during which Sb^3^⁺ was oxidized by atmospheric oxygen to Sb⁵⁺. The final white product obtained after thermal treatment was characterized and used for all subsequent studies.

### Computational Method

All first‐principles calculations were performed using density functional theory (DFT), as implemented in the Vienna Ab initio Simulation Package (VASP).^[^ ^40^, ^41^ ^]^ The projector augmented wave (PAW) pseudopotential method and a plane‐wave basis set were employed to describe the ionic cores and valence electrons, respectively. All structures in this study were fully relaxed until the Hellmann‐Feynman forces on all atoms were less than 0.02 eV/Å and the total energy convergence criterion was set to 10^−^⁶ eV. To ensure consistency, a plane‐wave cutoff energy of 520 eV and a k‐point mesh of 2 × 9 × 3 was used for all calculations. For structural optimization, the GGA+U method (Ueff = 2.5 eV) ^[^ ^42^ ^]^ was applied to account for strong electron correlations. The modified Becke–Johnson (mBJ) ^[^ ^43^ ^]^ potential was adopted for band structure calculations to improve the accuracy of electronic properties.

## Conflict of Interest

The authors declare no conflict of interest.

## Supporting information



Supporting Information

## Data Availability

The data that support the findings of this study are available from the corresponding author upon reasonable request.
