# Supplementary material for: Defect‐Free Sb‐Doping in Bi2O2Se Achieves Two‐Order‐of‐Magnitude Reduction in Saturation Intensity While Preserving High Carrier Mobility
Source: Adv Sci (Weinh). 2025 Nov 19;13(7):e18454. doi: 10.1002/advs.202518454 (PMC12866701; doi:10.1002/advs.202518454)
Supplement: Supplementary file 1 — Supporting Information [file ADVS-13-e18454-s001.docx]

Supporting Information

**Defect-Free Sb-Doping in Bi_2_O_2_Se Achieves Two-Order-of-Magnitude Reduction in Saturation Intensity While Preserving High Carrier Mobility**

Qingling Tang, Zhongben Pan*, Hongwei Chu, Han Pan, Dechun Li*

School of Information Science and Engineering and Key Laboratory of Laser and Infrared System of Ministry of Education

Shandong University

Qingdao 266237, China

E-mail: zhongbenpan@sdu.edu.cn; dechun@sdu.edu.cn


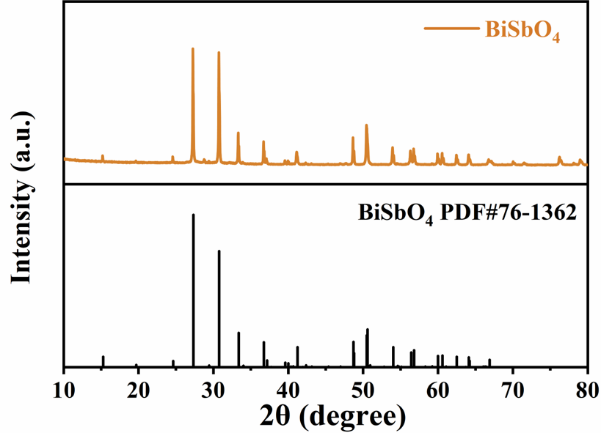


**Figure S1.** XRD of BiSbO_4_


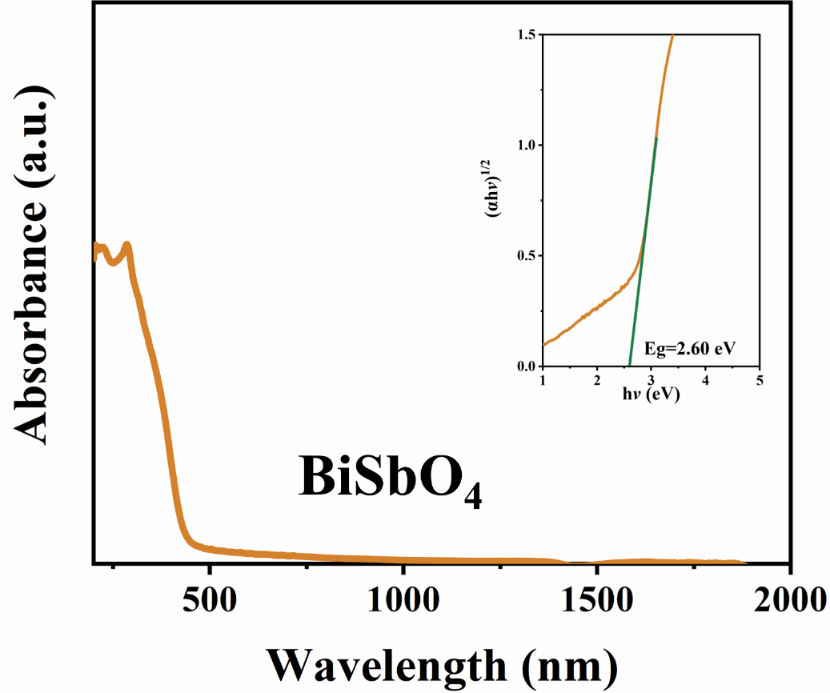


**Figure S2.** UV−VIS−IR absorption spectra of BiSbO_4_


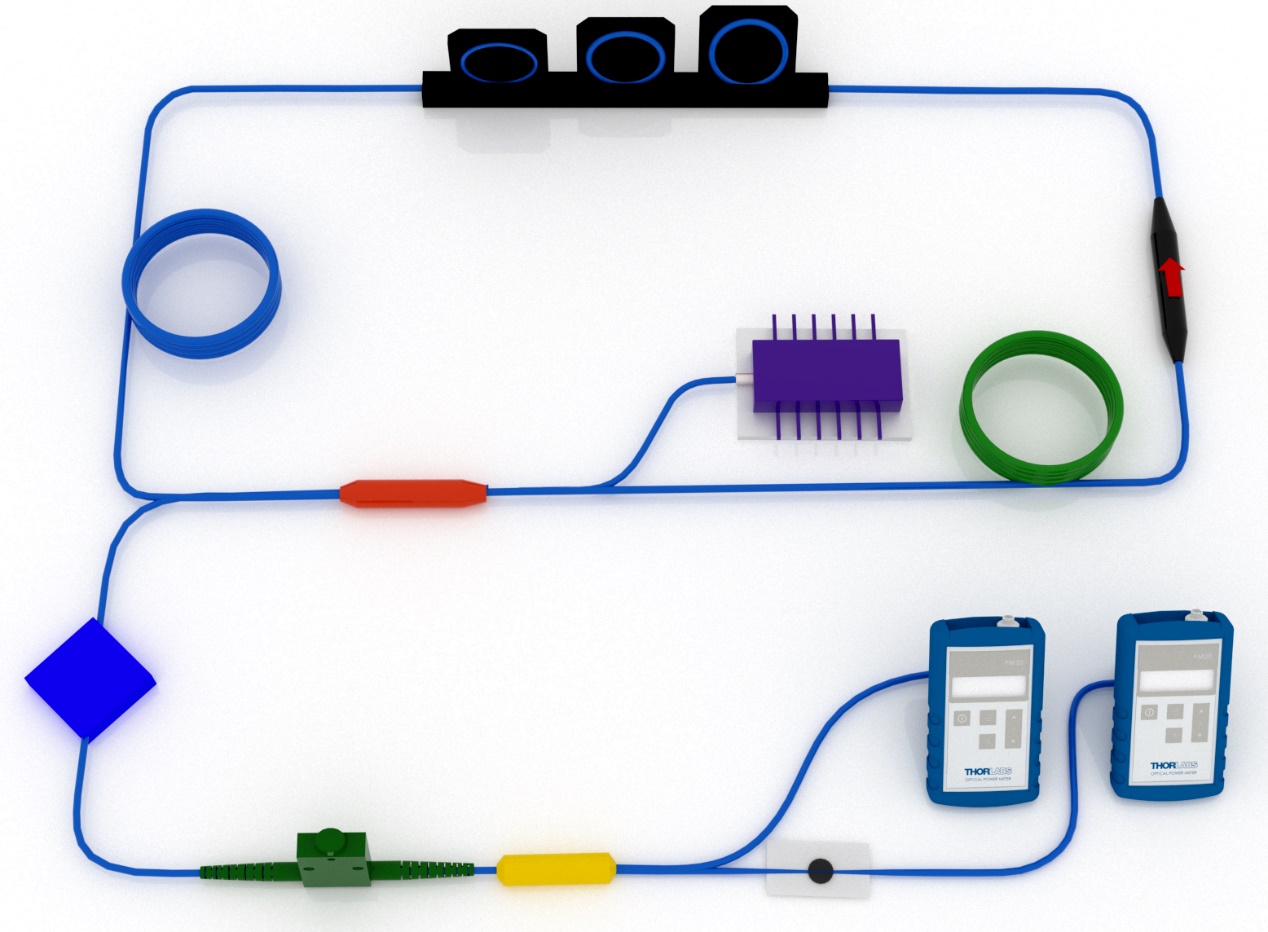


**Figure S3.** Schematic diagram of a dual-balanced detection system

The laser source consists of a custom-made nonlinear polarization rotated (NPR) Er-doped fiber laser, with center wavelengths of 1534 nm, and. The repetition frequencies are 11.17 MHz, and the pulse widths are 492 fs. The NPR mode-locked fiber laser consists of several key components, including a 976 nm laser diode (LD), a wavelength division multiplexer (WDM), a polarization controller (PC), a polarization-dependent isolator (PD-ISO), gain fibers (Er-doped fibers), and single-mode fibers (SMF-28e). To ensure that the saturable absorber receives sufficient laser power during testing, erbium-doped fiber amplifiers (EDFA) are used to further enhance the laser intensity. After amplification in the fiber amplifier, the laser intensity is adjusted using a variable optical attenuator (VOA) and split into two equal-intensity beams by a 1:1 optical coupler. One beam serves as the reference for power meter calibration, while the other propagates through the saturable absorber and is detected as the signal beam by the power meter.


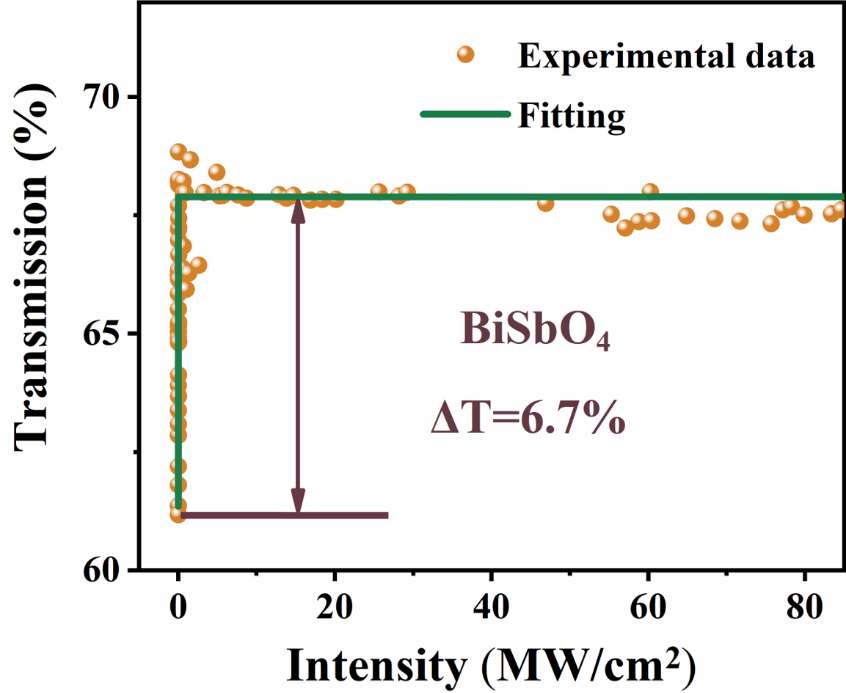


**Figure S4.** The nonlinear transmittance curves of BiSbO₄ at 1.5 μm.

**
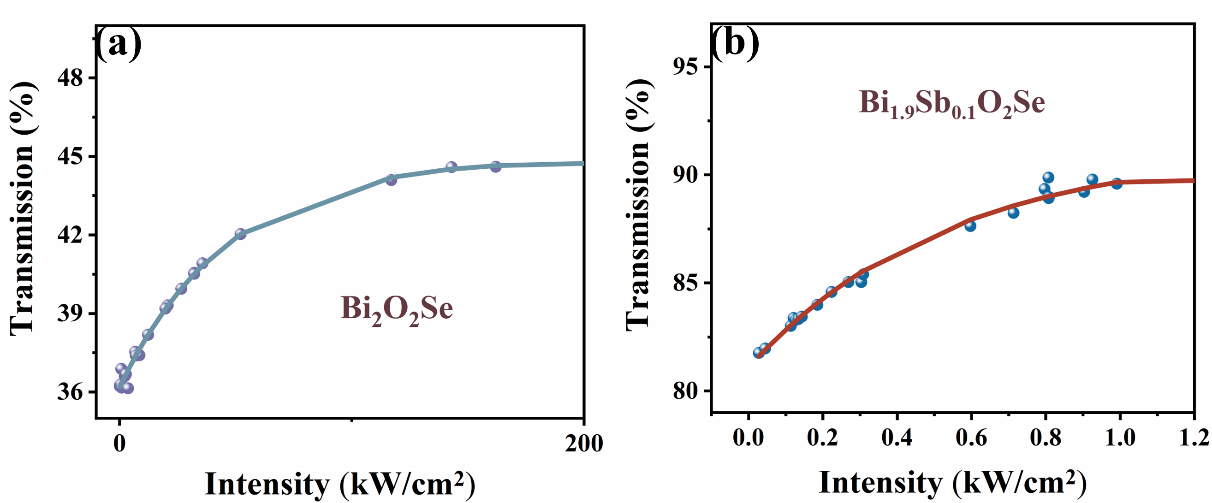
**

**Figure S5**. (a) The small-scale nonlinear transmittance curves of Bi_2_O_2_Se at 1.5 μm. (b)The small-scale nonlinear transmittance curves of Bi_1.9_Sb_0.1_O_2_Se at 1.5 μm.


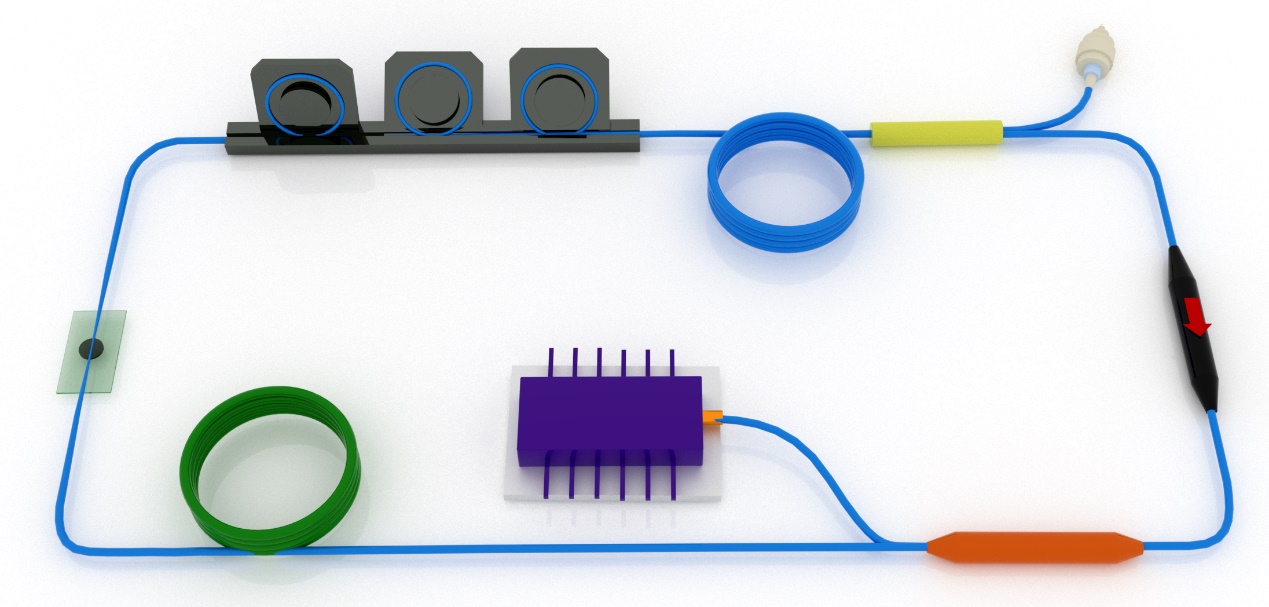


**Figure S6.** Experimental setup of a mode-locked fiber laser.

The EDFL was constructed using a conventional ring resonator design, which included a 0.3 m Er-doped fiber (Er110-4/125), 16.9 m of SMF (SM28e), a PI-ISO, a PC, a WDM (980/1550 nm), and an OC with a 10% tap ratio at 1550 nm. The 976 nm LD delivered a maximum output power of 450 mW, and the total cavity length of 19.3 m provided a net anomalous dispersion of -0.430 ps^2^.


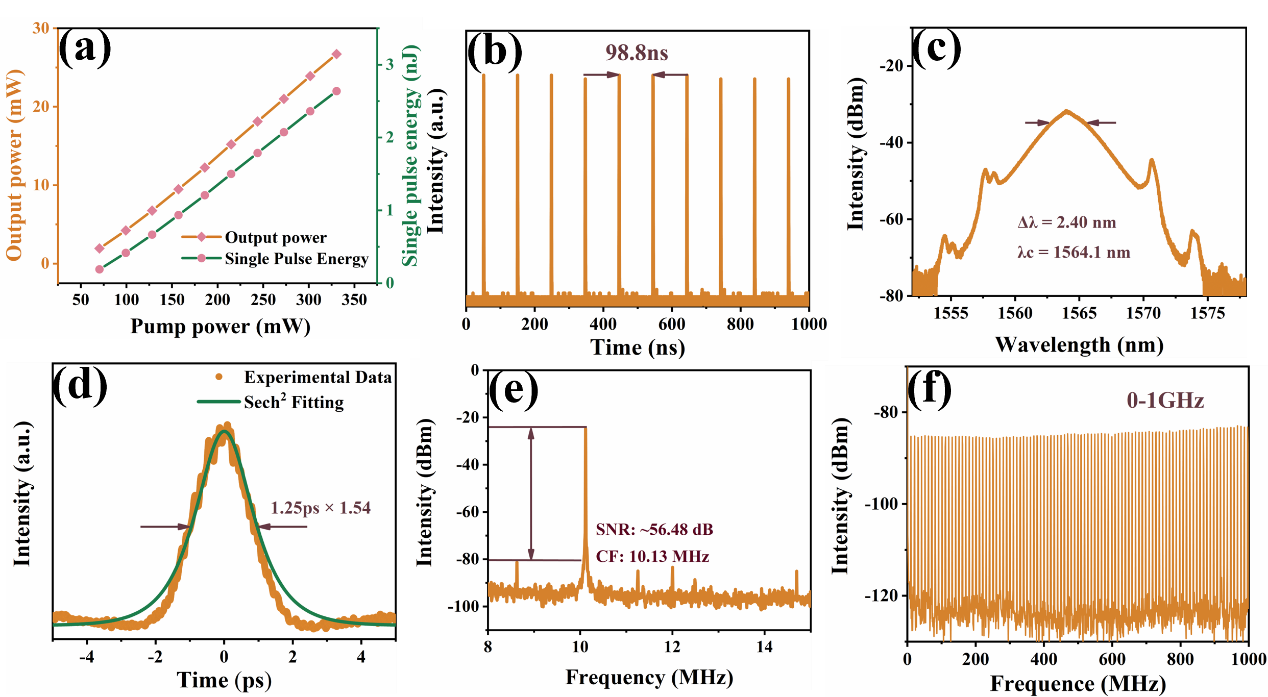


**Figure S7.** The mode-locking pulse output characteristics of BiSbO_4_ at 1.5 μm. (a) Variation of output power and single pulse energy. (b) Pulse sequence. (c) Optical spectrum. (d) Autocorrelation trajectory. (e) RF spectrum. (f)RF spectrum within a span of 0–1 GHz.

Figure S7(a) displays the linear relationship between output power, single-pulse energy, and pump power. As the pump power increased from 70.37 mW to 330.49 mW, the output power rose from 1.93 mW to 26.71 mW, corresponding to a single-pulse energy increase from 0.19 pJ to 2.64 nJ. Figure 7(b) shows a typical pulse train with a 1 μs time span and a pulse interval of 98.8 ns, matching a total cavity length of 20.3 m. Figure 7(c) presents the conventional soliton mode-locking spectrum centered at 1565.6 nm, with a 3 dB spectral width of 2.40 nm. The presence of distinct Kelly sidebands confirms the laser's operation in the conventional soliton mode-locking regime. The spectral asymmetry can be attributed to Raman self-frequency shift, soliton-dispersive wave interactions, and asymmetric gain spectra. As shown in Figure 7(d), hyperbolic secant fitting of the autocorrelation trace yielded a pulse width of 1.25 ps. Figure 7(e) shows the radio frequency (RF) spectrum with a central frequency of 10.57 MHz and a signal-to-noise ratio (SNR) of ~56.48 dB. Figure 7(f) is the RF spectrum over a 1 GHz span.

**Table S1.** The ICP-MS of of Sb-Bi_2_O_2_Se

| **Element** | **Atomic (%)** |
| --- | --- |
| Sb | 2.96 |
| Bi | 55.03 |

**Table S2.** Bond lengths around doping sites before and after Sb doping

|  | Bi₂O₂Se | Bi₁.₉Sb₀.₁O₂Se |
| --- | --- | --- |
| Bi(Sb)-O1 | 2.36 | 2.15 |
| Bi(Sb)-O2 | 2.36 | 2.22 |
| Bi(Sb)-O3 | 2.36 | 2.22 |
| Bi(Sb)-O4 | 2.36 | 2.15 |
| Bi1-O1 | 2.36 | 2.39 |
| Bi1-O2 | 2.36 | 2.39 |
| Bi2-O1 | 2.36 | 2.43 |
| Bi2-O3 | 2.36 | 2.43 |
| Bi3-O2 | 2.36 | 2.43 |
| Bi3-O4 | 2.36 | 2.43 |
| Bi4-O3 | 2.36 | 2.39 |
| Bi4-O4 | 2.36 | 2.39 |
